# Incidence and Cost of Rotavirus Hospitalizations in Denmark

**DOI:** 10.3201/eid1306.061432

**Published:** 2007-06

**Authors:** Thea Kølsen Fischer, Nete Munk Nielsen, Jan Wohlfahrt, Anders Pærregaard

**Affiliations:** * Statens Serum Institut, Copenhagen, Denmark; † Hvidovre University Hospital, Hvidovre, Denmark

**Keywords:** Rotavirus, children, epidemiology, economic burden, vaccination, research

## Abstract

In anticipation of licensure and introduction of rotavirus vaccine into the western market, we used modeling of national hospital registry data to determine the incidence and direct medical costs of annual rotavirus-associated admissions over <11 years in Denmark. Diarrhea-associated hospitalizations coded as nonspecified viral or presumed infectious have demonstrated a marked winter peak similar to that of rotavirus-associated hospitalizations, which suggests that the registered rotavirus-coded admissions are grossly underestimated. We therefore obtained more realistic estimates by 2 different models, which indicated ≈2.4 and ≈2.5 (for children <5 years of age) and ≈4.9 and ≈5.3 (for children <2 years of age) rotavirus-associated admissions per 1,000 children per year, respectively. These admissions amount to associated direct medical costs of US $1.7–1.8 million per year. Using 2 simple models to analyze readily available hospital discharge data resulted in more consistent and reliable estimates.

## Abstract 2

Two models provide reliable estimates that can be used to assess vaccine need and effectiveness.

Rotavirus is the main cause of acute, severe, dehydrating diarrhea in infants and children throughout the world (*1*). Rotavirus disease incidence is similar worldwide, regardless of infrastructure and other levels of development (*2*), which suggests that traditional diarrheal disease control measures, such as safe water and improved hygienic standards, are inadequate. In industrialized counties, hospitalizations are often the most costly events associated with rotavirus disease and often constitute a major expense for national health budgets (*3*).

A recent outbreak of rotavirus diarrhea in a daycare center in Denmark demonstrated that even small outbreaks of rotavirus in childcare facilities can be associated with substantial expense on a personal and a public scale due to parental loss of work (*4*). A major strategy for control of rotavirus disease is prevention through vaccination. With 2 new rotavirus vaccine candidates almost ready for marketing (*5*), valid and updated data on rotavirus disease extent and circulating rotavirus strains are essential for several purposes: to address the need for disease prevention, to generate reliable data for vaccine cost-benefit/effectiveness assessments, and to establish a platform for disease surveillance to monitor the effectiveness of a future vaccine program.

Because the immediate focus for development of rotavirus vaccines has mainly been prevention of associated deaths in Asia and Africa, valid data exist to some extent from prospective disease surveillance studies in these regions. However, despite the considerable problems associated with hospitalization, including nosocomial transmission of rotavirus disease (*6**,**7*) and the cost of parental loss of work (*8*), few data exist regarding the epidemiologic features of rotavirus infection in industrialized countries.

For this study we used Danish National Patient Registry (NPR) data for all hospital admissions in Denmark since 1977 to address the epidemiology and cost of rotavirus hospitalizations in Denmark. On the basis of findings from other studies of rotavirus disease (*9**,**10*), we anticipated an underreporting of rotavirus among patients hospitalized with diarrhea. This underreporting is due to various factors, the most important of which seems to be that rotavirus testing is routinely conducted in only a few settings because the same therapy, regardless of test results, is prescribed: symptomatic treatment with fluid replacement. Therefore, testing is used mostly for differential diagnostic purposes or to establish a diagnosis during outbreaks or for immunocompromised persons for whom rapid intervention against other diarrhea agents (bacteria, parasites) is crucial. Also, rotavirus laboratory results often are available only after the average diarrhea patient has already been discharged, so these results rarely get recorded in the patient’s medical file.

The issue of underreporting is supported by a study in a major county hospital in Copenhagen, Denmark. The findings suggest that among children 3–36 months of age, rotavirus was responsible for ≈60% of all admissions due to diarrhea from December 1998 through May 1999 (*11*). In Denmark, rotavirus-associated hospitalizations occur with a marked seasonality, from January to June, and peak in March and April. So far, no other diarrhea-associated microbial agent with a similar seasonal pattern has been identified. We took advantage of the unique seasonality of rotavirus to obtain a more realistic estimate of rotavirus incidence among hospitalized children. We used modeling of the 11 years of registry data as well as indirect estimates from similar rotavirus disease burden studies. Finally, we used these estimates to assess the extent of severe rotavirus disease in Denmark and the associated direct medical costs.

## Materials and Methods

### Data Sources

The NPR contains information on hospitalizations for all reasons except psychiatric in Denmark since January 1977, including outpatient treatments since 1995. Information on date of admission, date of discharge, diagnoses, surgical procedures, and personal identification number is recorded for every hospitalized patient. Diagnoses were classified according to the World Health Organization International Classification of Diseases, version 10 (ICD-10) (*12*). To identify specific rotavirus infections, we extracted ICD-10 diagnosis DA080. To compute data on diarrhea, we extracted ICD-10 diagnoses DA000 to DA099 (Table).

**Table:** Diarrhea-associated hospitalizations, by cause, for children <5 years of age, Denmark, 1994–2004*

Diagnostic category	ICD-10 code	Hospitalizations				
		Total no. (%)	Annual average	Incidence/ 1,000 child-years	% 0–23 mo. of age	% Boys
Etiology unspecified						
Presumed infectious	A09	22,475 (69.6)	2,043	6.7	67.8	55.9
Presumed noninfectious	A085	15 (0.1)	–	–	66.7	46.7
Etiology specified						
Viral, nonspecified	A084, A083	6,916 (21.4)	628	1.9	67.8	54.5
Norwalk virus and adenovirus	A081, A082	62 (0.2)	6	–	77.4	58.1
Rotavirus	A080	1,309 (4.1)	119	0.36	79.1	56.4
Bacterial	A00-A05	1,415 (4.4)	129	0.39	63.5	55.9
Parasitic	A06, A07	88 (0.3)	8	–	44.3	56.8
Total		32,280 (100)	2,935	8.9	67.8	55.6

*ICD-10, International Classification of Diseases, version 10; –, too few records to analyze.

### Data Analyses

From the NPR we extracted information for all Danish children <5 years of age who were hospitalized during January 1994–July 2005 and had diarrheal disease (ICD-10 codes DA000–DA099) as their primary or secondary diagnosis. If several different specific diagnoses of diarrhea, together with the nonspecific diagnosis of diarrhea (A099), were reported during 1 episode of hospitalization, all the different specific diagnoses were counted as unique diagnoses, whereas the nonspecific diagnosis (A099) was ignored. The nonspecific diagnosis of diarrhea counted as a diagnosis only when the hospitalized patient was registered as having nonspecific diarrhea. If a person had been admitted for diarrhea several times, only episodes >7 days apart were included in the study. For children <2 and <5 years of age, we estimated hospitalization incidence rates per 1,000 person-years at risk by using age- and period-specific person-years at risk in the Danish population.

Other studies, mainly from the United States, have demonstrated how many rotavirus-associated hospitalizations are registered as other types of diarrhea (*9**,**13*). To achieve a more realistic estimate of the number of rotavirus admissions, we applied 2 different approaches based on the number of all-cause diarrhea admissions.

The first approach was an indirect method known as the Brandt estimation method (*14*), which uses external information on the proportion of rotavirus admissions among all-cause diarrhea admissions. In this approach, the monthly number of rotavirus-associated hospitalizations was estimated by multiplying the monthly number of all-cause diarrhea hospitalizations with the month-specific proportion of rotavirus infections identified at a Copenhagen County university hospital during 1977–1978 (*15*).

In the second approach, the number of monthly rotavirus-associated hospitalizations during the rotavirus season was estimated as the registered number of all-cause diarrhea admissions minus the expected level on the basis of the much lower average level of all-cause diarrhea admissions outside the season. The expected number was estimated by using a log-linear Poisson regression model; the dependent variable was the monthly number of all-cause diarrhea admissions outside the season. A Poisson regression model was used because the number of hospitalizations traditionally can be assumed to be Poisson distributed. The log-linear regression form means that the logarithm of the mean parameter for the dependent variable is modeled by a linear combination of the independent variables. Two factors were included in the model as independent variables. The first factor took into account the varying monthly number of children at risk; this was done by including the logarithm of the risk time as a known factor (an offset). The second factor was a secular trend to allow for changes in the incidence during the study period; this was done by including time (months) as a continuous variable. In other words, we applied a log-linear Poisson regression model with number of all-cause diarrhea admissions as the dependent variable and logarithm of risk and time as independent variables. This regression model, based on the level outside the rotavirus season, was then used to estimate the monthly expected number of all-cause diarrhea admissions during the rotavirus season; i.e., the model for outside the season was extrapolated to the rotavirus season. The monthly observed number minus the expected number of all-cause diarrhea admissions during the rotavirus season was taken as an estimate of the monthly rotavirus-associated hospitalizations Estimation was performed within the age groups 0, 1, 2, 3, and 4 years and subsequently summed to achieve the total for children <5 years. The approach was based on 2 assumptions: first, that all rotavirus-associated hospitalizations were registered correctly in the months of July through December, when rotavirus is nonseasonal, and second, that the excess admissions during the annual peak season of diarrhea, January to June, were attributed to a pathogen believed to drive the intraseasonal all-cause diarrhea hospitalizations. The pathogen in this instance is rotavirus because no other gastrointestinal pathogen has yet been identified with the same seasonality.

According to the Danish National Board of Health, the price per hospitalization for diarrhea <4 days is US $1,420 (8,248 Danish krones [DKK] at a November 2006 exchange rate of 583 DKK to US $100). If the hospitalization is extended >3 days, cost is US $277 (1,608 DKK) per 24 hours of added stay (*16*). The costs include all expenditures related to the hospitalization (e.g., hospital bed, healthcare personnel, diagnostic testing, antimicrobial drugs, rehydration treatment, and intensive care). The costs are total costs and cannot be segregated further into the various above-listed expenditure categories (*16*). In Denmark, public hospital healthcare is free of charge. No private alternative is available for hospitalization of children with diarrhea.

## Results

### Epidemiology of All-Cause Diarrhea and Rotavirus-coded Hospitalizations

**Figure 1:**
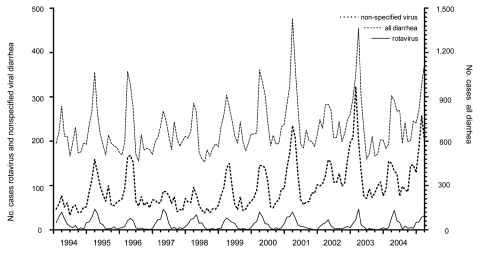
Monthly frequency of diarrhea-associated hospitalizations of children <5 years of age, Denmark, 1994–2005.

**Figure 2:**
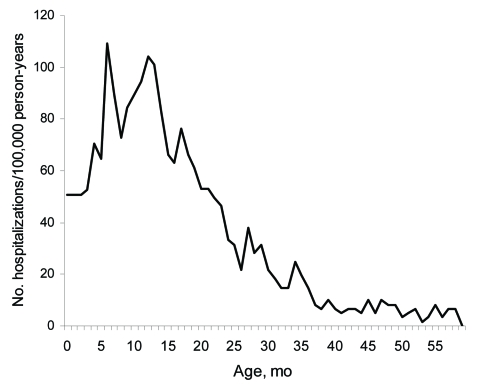
Incidence of rotavirus-coded hospitalizations in children <5 years of age, Denmark, 1994–2005.

We found a total of 32,280 unique diarrhea-associated hospitalizations in Denmark among children <5 years of age from 1994 through 2004. Slightly more boys (55.4%) than girls were hospitalized with diarrhea; median age was 16 months. The number of hospitalizations, regardless of diarrhea agent, remained relatively constant over time (Figure 1). A total of 1,309 admission records (annual average ≈120) contained the rotavirus-specific ICD-10 code; proportions by sex were 56% boys and 44% girls (Table). In children <5 years of age, 79% of admissions for rotavirus had occurred before the age of 2, compared with only 68% of admissions for all-cause diarrhea. Incidence rates of rotavirus-coded admissions peaked twice during early childhood, at 7 and 12 months of age (Figure 2).

**Figure 3:**
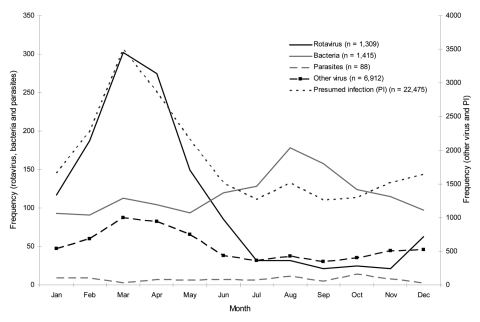
Seasonality of diarrhea-associated hospitalizations of children <5 years of age, Denmark, 1994–2005.

Viral infections constituted 85% of diarrhea-associated hospitalizations for which etiology was specified (Table). When studying the frequency of the main ICD-10 gastrointestinal disease categories (bacteria, virus, parasites, and presumed infectious) according to month, the seasonality of admissions coded as presumed infectious disease and virus without etiology each showed a seasonal pattern very similar to that of rotavirus, whereas admissions due to bacteria and parasites showed a more linear pattern throughout the year and bacterial infections showed a tendency to peak in the late summer and fall months of August through October (Figure 3). When comparing the trends for viral admissions without specified pathogen and trends for rotavirus admissions, we observed parallel seasonal trends throughout the entire study period (Figure 1). In all, 85% (1,115/1,309) of all rotavirus admissions and 63% (4,342/6,916) of nonspecified viral infections occurred from January through June, and the monthly numbers of admissions for these disease categories during the study period were significantly correlated (Spearman correlation coefficient 0.39, p = 0.007).

### Estimates of Rotavirus-associated Hospitalizations and Direct Medical Costs

Using the Brandt indirect method, we estimated that ≈840 hospitalizations (28.7% of all diarrhea) annually were due to rotavirus. This finding corresponds to ≈2.5 rotavirus-associated admissions per 1,000 children <5 years of age and ≈5.3 per 1,000 children <2 years of age annually. By using Poisson regression, we estimated that ≈780 annual hospitalizations (≈26.5% of all admissions for diarrhea in children) were associated with rotavirus, resulting in ≈2.4 and ≈4.9 annual rotavirus-associated admissions per 1,000 children <5 years of age and <2 years of age, respectively. The estimate was only slightly affected by defining the rotavirus season as January through June. Adding December to the season increased the estimate by 24 annual hospitalizations, and removing June from the season decreased the estimate by 8 annual hospitalizations.

During 1994–2004, 82.4% of all-cause diarrhea admissions lasted <3 days (n = 26,586), and 17.6% (n = 5,694) lasted >3 days (range 4–30 days). Thus, the median cost for an all-cause diarrhea admission was US $1,375 (8,013 DKK), and total cost for 2,935 annual admissions was therefore ≈US $4.6 million (≈27.1 million DKK) per year.

In terms of duration, 63% of rotavirus-associated hospitalizations lasted <3 days; 21%, 4–6 days; 9%, 7–13 days; and 7%, >13 days. By using the Poisson regression model estimate of 780 annual rotavirus-associated hospitalizations, we found the total direct medical cost of rotavirus hospitalizations to be ≈US $1.7 million (≈9.9 million DKK) per year. By using the indirect method estimate of 840 annual rotavirus-associated hospitalizations, we found the direct medical costs to be US $1.8 million (≈10.4 million DKK).

## Discussion

A decision to introduce new rotavirus vaccines into Denmark and most other European countries is likely to be based on a vaccine’s ability to protect against severe disease and prevent hospitalizations. Due to a combination of underreporting, low prevalence of testing for rotavirus, and misclassification, hospital episode statistics are rarely sufficient for assessment of the extent of national rotavirus-associated disease. Studies from the United Kingdom (*17*) and United States that used hospital discharge data have shown how the proportion of children coded with the specific rotavirus disease code often represents a gross underestimate (*18*). These observations can be explained in part by the combination of poorly defined criteria at most hospitals for requesting rotavirus testing and the fact that physicians’ diagnostic objective is often differentiation between the nosocomial highly active transmitting rotavirus and other less infectious agents for the purpose of isolation rather than therapeutic choices.

Our age-specific analyses demonstrated the magnitude of severe rotavirus infections among infants and young toddlers and showed disease peaks at 7 and 12 months of age. We believe the first peak is related to waning maternal antibody levels and the second peak to daycare attendance by Danish children (an effect of crowding and highly infectious transmissible environments).

Studies of seasonal trends of diarrhea-associated hospitalizations have shown that hospitalizations coded as diarrhea of nonspecified viral origin as well as diarrhea of presumed infectious origin have a marked winter peak, which suggests incomplete registration of rotavirus admissions. Our 2 different models estimated the annual rotavirus-associated hospitalizations to be between 780 and 840. We find it likely that the true contribution by rotavirus to all diarrhea-admissions is somewhere in between these numbers—or even higher, as the study from the late 1970s showed that rotavirus was identified among 37% of children admitted at a major Danish county hospital (*15*). A more recent estimate suggests that rotavirus infection constitutes an even higher proportion of diarrhea cases, ≈60% during the rotavirus seasonal months of December through April, but these data are based on a limited sample size of 69 (*11*). An updated prospective study of rotavirus and other diarrhea pathogens among children in Denmark is needed to further specify this estimate.

Despite underreporting and misclassification of rotavirus cases that results in gross underestimation of rotavirus disease, the coding of rotavirus diagnosis is relatively stable over time. The NPR system could be used as a timely and relatively sensitive tool by which to monitor the effectiveness of rotavirus vaccines. However, doing so would require improvements such as complete registrations of rotavirus infections, validation of diagnoses, and implementation of national guidelines for rotavirus sample collecting and testing.

A routine, universal rotavirus immunization program with a vaccine that is 75% effective against infection would prevent ≈45,000 cases of diarrhea annually among Danish children. If effectiveness were 95% against hospitalization, ≈700–800 hospitalizations could potentially be avoided per year, resulting in direct medical cost savings of ≈US $1.6 million. This estimate includes neither the number of nosocomial transmissions or outpatient visits prevented nor the indirect costs incurred when parents are forced to stay home from work to take care of sick children, factors that in western societies are likely more influential than the problem of hospitalization alone (*19*). Weighing the health benefits of vaccination against its costs requires a measure like quality-adjusted life years, which takes both reduced illness and death into account, and subsequent cost-effectiveness analyses in which the healthcare-associated as well as the societal costs are considered.

Our study provides updated information on the extent of disease and the cost of diarrhea- and rotavirus-specific hospitalizations in a European country. These data are often requested by health officials (*20*) to help increase the awareness of rotavirus disease in Europe and help health officials assess the potential benefits of disease prevention through vaccination.
